# Low titer group O whole blood inventory conservation: The effect of implementing group A whole blood at a simulated civilian trauma center

**DOI:** 10.1111/trf.70235

**Published:** 2026-04-17

**Authors:** Emilie A. Jochumsen, Jennifer Gurney, Donald Jenkins, Jansen N. Seheult, Philip C. Spinella, Ulrik Sprogøe, Mark H. Yazer

**Affiliations:** ^1^ Department of Clinical Immunology Odense University Hospital Odense Denmark; ^2^ Department of Surgery Brook Army Medical Center and The Joint Trauma System San Antonio Texas USA; ^3^ Department of Surgery UTHealth San Antonio San Antonio Texas USA; ^4^ Division of Hematopathology Mayo Clinic Rochester Minnesota USA; ^5^ Trauma and Transfusion Medicine Research Center, Department of Surgery University of Pittsburgh Pittsburgh Pennsylvania USA; ^6^ Department of Critical Care Medicine University of Pittsburgh Pittsburgh Pennsylvania USA; ^7^ Department of Pathology University of Pittsburgh Pittsburgh Pennsylvania USA

**Keywords:** civilian, group A, hospital, inventory, low titer group O whole blood, management, simulation

## Abstract

**Background:**

Substituting group A whole blood (GAWB) might relieve some of the burden on the low titer group O whole blood (LTOWB) inventory. This simulation examined the effect of implementing GAWB at a large trauma hospital.

**Study Design and Methods:**

A civilian trauma center with LTOWB and GAWB par levels set to 22 and 10 units, respectively, was modeled as the baseline. The average daily number of WB recipients was 0.5 who received an average of four WB units per patient. LTOWB was issued in packages of five and all had to be consumed before a group A patient could be switched to GAWB. Several variations on the baseline simulation were also modeled.

**Results:**

In the baseline simulation, there was a median (interquartile range, IQR) of 1 (0–2) GAWB and 16 (13–19) LTOWB recipients per month. The median (IQR) GAWB and LTOWB units transfused per month was 2 (0–9) and 56 (42–72), respectively. Fourteen percent of group A patients received any quantity of GAWB. The variations that had the most impact on GAWB usage included increasing the number of WB recipients to 2.5 per day where the median (IQR) monthly number of GAWB recipients and units transfused increased to 4 (3–5) recipients and 21 (12–29) units, respectively, and when the average WB requirements increased to eight units per patient resulting in transfusing a median (IQR) of 10 (5–15) GAWB units per month to 3 (1–4) recipients.

**Discussion:**

Under routine civilian hospital circumstances, implementing GAWB will have a modest impact on LTOWB inventory.

**Abbreviations**
CPG: clinical practice guideline
GAWB: group a whole blood
IQR: interquartile range
LTOWB: low titer group O whole blood
RBC: red blood cell
WB: whole blood

## 1 INTRODUCTION

The use of low titer group O whole blood (LTOWB) is increasing in the United States for the treatment of patients in hemorrhagic shock from both trauma and non‐trauma etiologies.^1^, ^2^, ^3^ However, concerns have been raised about the effect on the group O red blood cell (RBC) inventory when eligible group O donors are shunted toward LTOWB donation and away from donating whole blood (WB) that could be used to manufacture components.

While computer models suggest that the effect of collecting LTOWB on the group O RBC inventory is minimal, especially for centers with a large number of collections,^4^ one proposed solution to this hypothetical problem is to use group A WB (GAWB) for patients who are confirmed to be group A and in need of ongoing balanced resuscitation. Patients would begin their resuscitation with LTOWB, and once they were confirmed to be group A, they could be switched to GAWB for the remainder of their WB transfusion needs. Patients whose ABO group is already known, such as those with scheduled elective surgeries or those who are in‐patients with a valid type and screen, could start their resuscitation with GAWB instead of LTOWB. Using GAWB could conserve some LTOWB units for use by non‐group A recipients and could reduce the number of group O donors who donate LTOWB, thereby relieving some of the burden on the group O RBC inventory.

GAWB donors would not need to have low titer anti‐B because its use will be group‐specific, although all of the other eligibility criteria for donating WB would still need to be met. The US Army recently published a clinical practice guideline (CPG) that allows for GAWB use only at in Role 3 (the highest level of care on the battlefield) deployed medical facilities, where a transfusion laboratory is available to perform ABO groupings; this should be considered so as to help preserve the LTOWB inventory in this austere surgical environment.^5^

However, the main limitation of implementing GAWB is the speed at which the patient's ABO group can be confirmed because until that happens, LTOWB and other uncrossmatched universally compatible products should be administered. Also, the majority of recipients, approximately 60%, will not be group A, thereby limiting the ability of GAWB to relieve the use of LTOWB.

The aim of this study was to evaluate GAWB and LTOWB use and wastage, using a simulated hospital model based on clinical practice assumptions from large American trauma centers.

## 2 MATERIALS AND METHODS

### 2.1 Modeling a hospital that uses WB for resuscitating patients in hemorrhagic shock

First, a baseline simulation was constructed using the assumptions in Table 1. Then, six variations on this simulation were performed to determine their effect on GAWB utilization and wastage.

**TABLE 1:** Assumptions for the blood bank and whole blood recipients for the baseline simulation.

	Variable	Assumption
Blood bank variables	Par level of LTOWB units	22
	Par level of GAWB units	10
	Age of WB units at beginning of simulation	Normal distribution around 10 days, truncated at 2 days (i.e., 2–20 days)
	LTOWB and GAWB unit replenishment schedule	If ≥75% of par level left, replenish to 100% of par level
		If 50–74% of par level left, replenish to 75% of par level
		If <50% of par level left, replenish to 50% of par level
	Expiration of LTOWB & GAWB	21 days
	Age of LTOWB & GAWB upon replenishment	Randomly selected between 2 and 4 days old
	Number of LTOWB units in MTP	5 units
	WB issuing strategy	Oldest units issued first
Patient/hospital variables	ABO distribution of recipient	O 45%, A 40%, B 10%, AB 5%
	Number of daily WB recipients	0 patients (60%) 1 patient (30%) 2 patients (7%) 3 patients (2%) 4 patients (1%)
	Number of WB units needed per patient	22% of patients needed 1 unit 30% of patients needed 2 units 13% of patients needed 3 units 8% of patients needed 4 units 5% of patients needed 5 units 2% of patients needed 6 units 2% of patients needed 7 units 3% of patients needed 8–10 units (randomly sampled) 4% of patients needed 11–20 units (randomly sampled) 2% of patients needed 21–30 units (randomly sampled) 1% of patients needed 31–40 units (randomly sampled)
	Number of units administered before ABO group confirmed	0 units 5% of patients 1 unit 5% of patients 2 units 5% of patients 5 units 30% of patients 10 units 30% of patients 15 units 15% of patients 20 units 10% of patients

Abbreviations: GAWB, group A whole blood; LTOWB, low titer group O whole blood; MTP, massive transfusion protocol; WB, whole blood.

To achieve an average of 0.5 WB users per day, the model selected between zero to four patients to receive WB based on the frequency distributions listed in Table 1, with a maximum of four patients presenting to the hospital in hemorrhagic shock on 1% of the simulated days. The total number of WB units that each patient was assigned to require was also determined by randomly selecting a quantity within the frequency ranges listed in Table 1. Based on this frequency, the average number of WB units required per patient was four units.

In the baseline simulation, the recipient's ABO group became known after a randomly assigned number of LTOWB units had been transfused, drawn from a weighted discrete distribution (Table 1). This variation in the amount of time until the recipient's ABO group was known accounted for the variation in how long it can take before an actual blood bank receives a sample on a patient and can confirm their ABO group. Only after the recipient's ABO group was confirmed could a group A recipient be switched to GAWB. In the baseline simulation, the ABO group of 5% of the WB recipients was known at the time that their resuscitation began, that is, after zero units of LTOWB had been transfused. This replicated the situation where a patient's ABO group was known before they started bleeding such as a patient undergoing scheduled surgery where a type and screen had been ordered ahead of time. Such a patient could immediately be provided with GAWB, if they were group A.

For all patients, LTOWB units were issued five units at a time, which replicates the issuing of LTOWB in a massive transfusion protocol (MTP) packet. Thus, for some patients the amount of LTOWB issued would greater than what would be needed. In the baseline simulations and in all variations, there would be a 1% chance that LTOWB units that had been issued but not transfused, that is, “orphaned”, would be lost and counted as “wasted.” All patients, except those who were known to be group A when their resuscitation began, would thus receive at least one unit of LTOWB.

In the baseline simulation, group A patients would only be able to receive GAWB after all LTOWB units in the issued MTP(s) had been transfused. For example, a group A patient randomized to need eight units of WB, whose blood type became known after two LTOWB units had been transfused, would receive the five LTOWB units issued in the initial MTP and then three GAWB units. If this patient's blood type became known after 10 units, all eight units would have been LTOWB, as two MTPs would be issued. This replicates the situation where it is not possible to immediately start providing a group A patient with GAWB because in reality, switching blood products midway through a resuscitation can take some time during which additional LTOWB units are transfused (see variation 1 below).

### 2.2 Modeling the hospital blood bank

The blood bank's LTOWB inventory par was set at 22 units, which was the 95th quantile of the WB demand of all of the simulated patients combined plus an additional five units. The 95th quantile was determined by a simulation of 20,440 days using the distribution of WB demand shown in Table 1. This par level was selected to allow the blood bank to meet the LTOWB demand of the 95th quantile of patients and still be able to issue one more MTP should another patient arrive before the inventory could be resupplied. As GAWB can only be issued to a select group of patients, the inventory par was set at a lower level of 10 units.

For all simulations, the initial ages of the LTOWB and GAWB units in the blood bank's inventory were randomly assigned over a normal distribution centered on 10 days; however, the low‐end of the units' ages was truncated at two days because receiving units that are zero or one day old is improbable. All WB units had an expiration of 21 days. Older units were always issued before fresher units.

LTOWB and GAWB inventories were resupplied at two time points every day if the inventory levels were below their respective par levels. On days when multiple patients were randomized to receive WB, the arrival and transfusion of the patients relative to the resupply time points were randomized. However, the final resupply of the day always occurred after all patients had been transfused. The final resupply of the day also replenished units that would expire the next day. The number of expired LTOWB and GAWB units per day was included in the “wasted” category.

The amount of units restocked per resupply was calculated based on the WB inventory's deviation from its par. For both LTOWB and GAWB, when the inventory before resupply was less than 50% of the par, the inventory would be resupplied up to 50% of the inventory par; when the inventory was between 50 and 74% of its par, it would be resupplied to 75%; when the inventory was more than 74% of its par, the inventory would be resupplied to 100% of the par (Table 1). Rounding of the resupply fraction could theoretically resupply the inventory to 23 LTOWB units. The ages of the replenished WB units were randomly selected between 2 and 4 days old.

### 2.3 Additional simulation variations

To simulate hospitals with different characteristics compared to the baseline simulation hospital, six variations of the baseline simulation were performed, where one variable was altered per variation (Table 2):

1. Switch before the LTOWB in the MTP is completely used: In this variation, group A recipients were immediately switched to GAWB as soon as their ABO type was confirmed. This represents the optimal scenario in terms of utilizing GAWB for eligible patients and conserving LTOWB. For example, a group A patient randomized to need five units of WB and whose blood type became known after two LTOWB units had been transfused would have received two LTOWB units and three GAWB units. In this example, three LTOWB units from the initial MTP would not have been transfused and would have become “orphaned” with a 1% chance of being wasted.
2. GAWB inventory par decreased from 10 units to 4 units: The GAWB inventory par was reduced to four units in order to model the reduction in GAWB wastage that would naturally occur if fewer GAWB units were kept in inventory.
3. Increase in the mean number of WB recipients from 0.5 to 2.5 per day: The frequency distribution of the daily number of WB users was adjusted so that the average was increased from one patient every second day to five patients every second day. The altered frequency distribution for this variation is listed in Table 2.
4. Faster recipient ABO type confirmation: This variation simulated a hospital that could confirm the recipient's ABO type faster than the baseline hospital thereby potentially decreasing the time required to start providing GAWB units. This was achieved by changing the frequency distribution to the distribution listed in Table 2.
5. Increased proportion of patients with known pre‐resuscitation ABO types: In this variation, the fraction of patients who could begin their resuscitation with GAWB because their ABO group was known before their resuscitation began was increased from 5% to 20%. The complete frequency distribution for this variation is listed in Table 2.
6. Increase in the mean number of WB units required per patient from 4 to 8 units: Increasing the WB demand per patient for this variation was achieved by using the distribution listed in Table 2.

**TABLE 2:** Variable alterations used for simulation variations.

	Variable altered	Altered assumption
**Variation 1**: Switch before all LTOWB in MTP is used	GAWB can be issued before all LTOWB in MTPs have been used	Simulation rule changed
**Variation 2**: GAWB par at 4 units	Par level of GAWB units	4 GAWB units
**Variation 3**: 2.5 WB patients per day	Number of daily WB recipients	0 patients (5%) 1 patient (10%) 2 patients (30%) 3 patients (30%) 4 patients (10%) 5 patients (5%)
**Variation 4**: Faster ABO typing	Number of units administered before ABO group confirmed	0 units 5% of patients 1 unit 5% of patients 2 units 15% of patients 5 units 40% of patients, 10 units 20% of patients 15 units 10% of patients 20 units 5% of patients
**Variation 5**: More known ABO types at start of resuscitation	Number of units administered before ABO group confirmed	0 units 20% of patients 1 unit 4% of patients 2 units 4% of patients 5 units 25% of patients 10 units 25% of patients 15 units 13% of patients 20 units 9% of patients
**Variation 6**: Increased WB blood demand from 4 to 8 units	Number of WB units needed per patient	1% of patients needed 1 unit 2% of patients needed 2 units 3% of patients needed 3 units 5% of patients needed 4 units 5% of patients needed 5 units 15% of patients needed 6 units 20% of patients needed 7 units 25% of patients needed 8 units 7% of patients needed 9 units 2% of patients needed 10 units 7% of patients needed 11–20 units (randomly sampled) 2% of patients needed 21–30 units (randomly sampled) 1% of patients needed 31–40 units (randomly sampled)

Abbreviations: GAWB, group A whole blood; LTOWB, low titer group O whole blood; MTP, massive transfusion protocol; WB, whole blood.

Note that variations 2–6 were performed with the model that required the transfusion of all of the issued LTOWB units before GAWB could be transfused because it is likely the most realistic scenario for handling two different inventories of WB.

In the baseline simulation and all of the variations, the intention was to administer enough WB to meet a patient's entire WB demand, once their blood type was confirmed. For example, a group A patient who was randomized to receive 15 units of WB in total and who received five units of LTOWB and only eight units of GAWB due to GAWB inventory limitations would have received two more units of LTOWB.

### 2.4 Simulation output

The objectives of this simulation were to determine how many GAWB units would be used assuming that each GAWB unit transfused saved an LTOWB unit, as well as the wastage rates of GAWB and LTOWB units. Also, the fraction of GAWB recipients among group A patients, the median age of transfused products, and the fraction of patients whose full WB needs could not be met was documented.

Each simulation was a loop of 365 days, iterated 56 times, thereby simulating a total of 20,440 days per simulation (baseline and the variations).

The simulations were written using base R (R Version 4.3.0) and the “dplyr” package.

## 3 RESULTS

**FIGURE 1:**
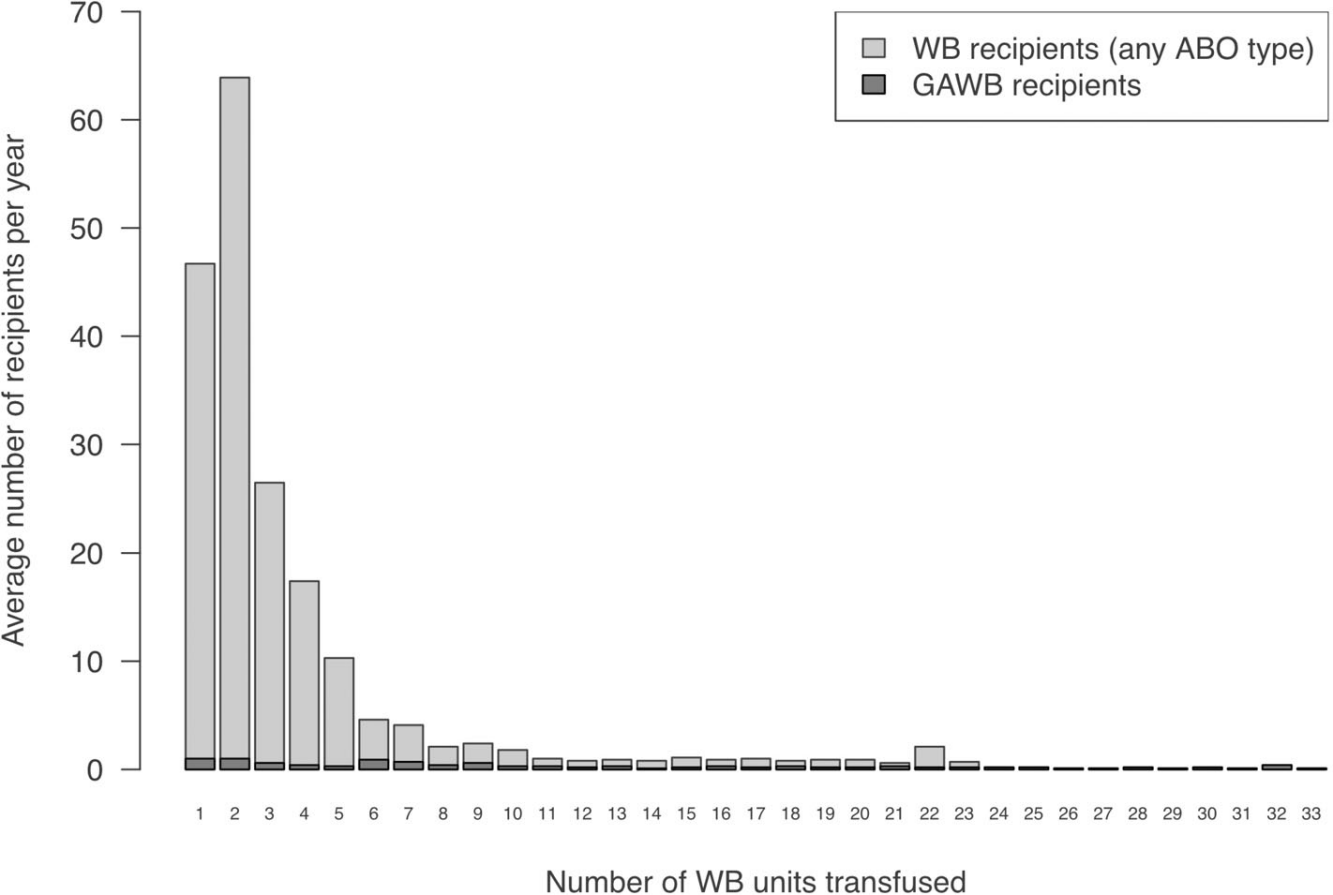
Distribution of whole blood (WB) units transfused and the average number of recipients per year. All recipients received at least one unit of WB; the group A whole blood (GAWB) recipients received at least one unit of GAWB.

**FIGURE 2:**
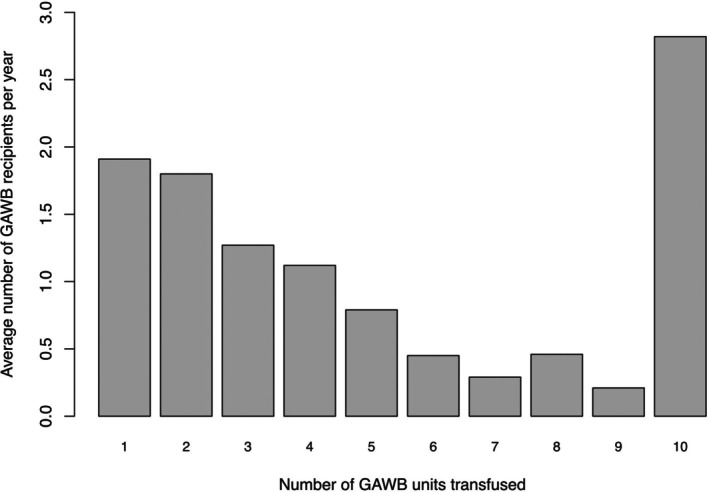
Distribution of group A whole blood (GAWB) units and the average number of recipients per year.

The results of the baseline simulation are shown in Table 3. The median (IQR) number of GAWB recipients per year was 11 (9–14). The right skewed distribution in Figure 1 meant that most patients received 1 to 2 units of WB, in order to achieve the average demand per patient of 4 units. Thus, the y‐axis of Figure 1 is shown in years so as to be able to demonstrate the small number of GAWB recipients among the WB recipients. Figure 1 also demonstrates that among the relatively few number of GAWB recipients, the combined quanta of WB transfused showed much greater variation compared to patients who only received LTOWB. Figure 2 demonstrates the number of GAWB units transfused per year. The most common quantum of GAWB units transfused was 10 units, which occurred for approximately three patients per year.

**TABLE 3:** Results of the baseline simulation and six variations.

Baseline simulation		Variation 1	Variation 2	Variation 3	Variation 4	Variation 5	Variation 6
		Switch before all LTOWB in MTP is used	GAWB par at 4 units	2.5 WB patients per day	Faster ABO typing	More known ABO types at start of resuscitation	Increased WB blood demand from 4 to 8 units
Median number of GAWB recipients per month [IQR]	1 [0–2]	1 [0–2]	1 [0–1]	4 [3–5]	1 [0–1]	1 [0–2]	3 [1–4]
Median number of LTOWB recipients per month [|IQR]	16 [13–19]	16 [13–19]	16 [13–19]	74 [69–78]	16 [13–19]	16 [13–19]	16 [13–19]
Median number of GAWB units transfused per month [IQR]	2 [0–9]	3 [0–10]	2 [0–4]	21 [12–29]	3 [0–10]	2 [0–9]	10 [5–15]
Median LTOWB units transfused per month [IQR]	56 [42–72]	55 [41–70]	58 [42–74]	244 [226–266]	55 [43–72]	55 [42–72]	112 [92–137]
Median number of GAWB units wasted per month [IQR]	13 [10–17]	13 [10–16]	4 [4–6]	7 [2–10]	13 [10–17]	13 [10–16]	10 [7–13]
GAWB waste rate (%) for the duration of the simulation [Wilson 95% CI]	74 [73–75]	72 [71–73]	65 [63–66]	23 [22–23]	72 [71–73]	73 [73–74]	48 [47–49]
LTOWB waste rate (%) for the duration of the simulation [Wilson 95% CI]	10 [10–10]	10 [10–11]	9 [9–10]	2 [1–2]	9 [9–9]	10 [10–10]	1 [1–1]
Fraction (%) of group A patients who received any quantity of GAWB [Wilson 95% CI]	14 [13–15]	18 [17–19]	14 [13–15]	14 [13–14]	15 [14–16]	17 [16–18]	42 [41–44]
Fraction (%) of patients with unmet WB needs [Wilson 95% CI]	3 [2–3]	3 [2–3]	3 [3–4]	7 [7–7]	3 [3–4]	3 [3–3]	6 [5–6]
Median age (days) of GAWB when transfused [IQR]	13 [9–18]	14 [8–18]	12 [7–17]	10 [6–15]	13 [9–18]	14 [9–18]	13 [8–17]
Median age (days) of LTOWB when transfused [IQR]	12 [8–16]	12 [8–16]	12 [8–16]	4 [4–5]	12 [8–16]	12 [8–16]	7 [5–10]

Abbreviations: CI, confidence interval; GAWB, group A whole blood; IQR, interquartile range; LTOWB, low titer group O whole blood; WB, hole blood.

The median number of GAWB units transfused per month was relatively small in the baseline simulation (1, IQR 0–2) and did not change in variation 1 where the recipient could be switched to GAWB before all of the issued LTOWB had been transfused (1, IQR 0–2; Table 3). Thus, the median monthly savings to the LTOWB inventory by using GAWB was negligible in this variation. The latter variation had the second highest fraction of group A patients who received any quantity of GAWB (18%), only surpassed by the variation where WB demand increased from 4 to 8 units (42%) (variation 6). The GAWB wastage rate was somewhat reduced in variation 2 when the GAWB inventory par level was decreased to four units (65%) compared to the wastage when the GAWB par level was 10 units in the baseline simulation (74%). Confirming the patient's ABO group faster (variation 4) did not significantly increase the median number of GAWB units transfused (1, IQR 0–1) compared to the baseline simulation (1, IQR 0–2). Interestingly, faster ABO confirmation did not have an impact on the percentage of group A patients who received any quantity of GAWB compared to the baseline simulation (15% vs. 14%, respectively). In fact, being able to start the resuscitation of group A patients with GAWB (variation 5) produced very similar GAWB usage parameters to the simulation where the recipient's ABO group was confirmed faster than the baseline simulation (variation 4) and to the baseline simulation.

In general, the greatest monthly number of GAWB units transfused, the lowest monthly GAWB wastage and youngest median age of the transfused WB units was observed in the simulation variations where the average number of WB daily recipients was increased from 0.5 to 2.5 (variation 3) and when the average WB demand was increased from 4 units to 8 units (variation 6). In both variations, the demand on the WB inventories increased drastically compared to the baseline simulation. This led to more frequent exhaustion of both WB inventories such that more than double the percentage of patients had unmet WB needs (7% in variation 3 and 6% in variation 6) compared to the baseline simulation (3%). The repeated inventory exhaustion is likely because the par levels for both inventories were unchanged from the baseline simulation.

## 4 DISCUSSION

These simulations revealed that the savings in LTOWB units from using GAWB for eligible patients are likely small unless demand for WB is very large. Intuitively it would seem that there would be significant savings in LTOWB units by using GAWB when possible. However, the low but realistic average number of daily WB patients (0.5 patients per day) and the mean quantity of WB required by these simulated patients (4 units per patient) did not permit the use of large quantities of GAWB during their resuscitation.

Variations 1, 4, and 5 were designed to test the effect of being able to more rapidly switch group A patients to GAWB compared to the baseline simulation. Yet, in spite of the ability to more rapidly provide GAWB, the median number of GAWB recipients per month was 1 in all of these variations. This low number of recipients combined with the facts that the majority of patients had a WB demand of fewer than 4 units and that many of their ABO groups became known after they had received at least the first MTP, that is, five LTOWB units, likely explains why the number of GAWB units transfused was low and GAWB wastage was high. Furthermore, to implement variation 1 where the clinical team could switch to GAWB before the entire quantity of LTOWB in the MTP had been transfused would likely involve issuing GAWB along with LTOWB. The blood bank would presumably then call the team and inform them when/if they can start using GAWB once the patient's ABO group is known. This is a risky procedure that would potentially increase the risk of an ABO‐incompatible mistransfusion.

It was interesting to note that nearly the same number of group A patients received either one or 10 GAWB units in the baseline simulation (Figure 2). Given the overall relatively low total quantity of WB that these patients required, it is not surprising that many group A patients only received one unit of GAWB in this simulation. However, for those patients with a large total WB requirement, it is not surprising that the entire GAWB inventory was transfused to one eligible patient. This further reinforces the finding that the largest impact of GAWB in terms of saving LTOWB units will be at institutions that treat high‐volume WB users and/or a high number of recipients.

Most hospitals in the United States, however, do not receive as many as 2.5 WB users per day (variation 3) and having an average blood demand per patient of 8 (variation 6) is unrealistically high for almost any civilian trauma center in the United States. These factors could, however, change depending on the settings or population. It would be interesting to perform this simulation using downrange military data, or at a hospital network that shares a common blood supply, where both of those parameters might be met more often than when modeling a single civilian trauma center.

In variation 2 where the GAWB inventory par was decreased to 4 units, the wastage decreased slightly compared to the baseline simulation where the par level was 10 GAWB units. This is interesting because decreasing the inventory by 60% only yielded an absolute wastage decrease of 9% (74%–65%) compared to the baseline simulation. This was likely caused by the fact that, while the number of GAWB recipients per month was low, many of these patients had a demand of 10 units or more (Figure 1).

This simulation has several limitations. The main limitation is that the findings and conclusions from these simulations apply to a hospital with the same practice patterns and blood bank stocking and issuing practices as this simulated hospital. However, the assumptions that underpinned the baseline and variation simulations were based on the experience from several large American trauma centers and should be applicable to many hospitals throughout the United States. If hospitals are not able to restock their blood supplies midway through the day, they would likely have set a higher WB inventory par level. The resupply schedule of this simulated hospital allowed for smaller LTOWB and GAWB inventories, which in theory would decrease the wastage of both products compared to a hospital that could not restock midway through the day. Furthermore, the low frequency of GAWB recipients would mean the GAWB inventory would likely be fully stocked most of the time, even if resupplies occurred at a slower pace. The percentage of par level to which the hospitals were resupplied twice daily was designed to incorporate the often limited WB inventory that a blood supplier might have available. Because of the supplier's potentially limited WB inventory, it might not always be possible to fully replete the hospital's WB inventory, so a graded restocking based on the hospital's current inventories of GAWB and LTOWB and their par levels was performed. Based on the results of the variations performed, it is unlikely that restocking the hospital's WB inventories to their maximum par levels would have increased utilization significantly while surely increasing wastage. The WB needs for these simulated patients do not necessarily indicate the entire quantity of blood products that were needed for their resuscitation and subsequent rebleeding, but modeling these was beyond the scope of the simulation. Lastly, it is important to consider that in this simulation (except for variations 4 and 5), 75% of the patients' ABO types were not known until after 10 LTOWB units had been transfused. However, transfusing such a quantity before the ABO type is confirmed is not uncommon due to the fact that most hospitals typically require a second ABO check type sample in patients without a historical ABO type before ABO‐specific products can be issued.

In this simulation, implementing a GAWB inventory to complement the LTOWB inventory provided only modest savings of LTOWB units. The factor that increased GAWB use the most, and therefore affected the greatest LTOWB savings, was increasing the daily number of WB recipients. Increasing the average WB use per patient also helped save LTOWB units, albeit to a lesser extent than increasing the daily number of recipients. Hospitals with relatively small volume and number of WB users should carefully consider the logistical and wastage implications of implementing a GAWB inventory for the purpose of LTOWB inventory conservation.

## CONFLICT OF INTEREST STATEMENT

P.C.S. consults for Cerus, is on the scientific advisory board for Haima, Grifols, and Octapharma, and is a co‐founder and chief medical officer for Kalocyte. M.H.Y. is on the scientific advisory board for Hemanext, consults for Legacy Innovations, has given paid lectures for Terumo BCT, Grifols, and owns equity in Velico Medical, Inc.

## Data Availability

Data sharing not applicable to this article as no datasets were generated or analysed during the current study.
